# Effects of Thickness of the Corn Seed Coat on the Strength of Processed Biological Materials

**DOI:** 10.3390/ma18020222

**Published:** 2025-01-07

**Authors:** Łukasz Gierz, Weronika Kruszelnicka, Wiktor Łykowski, Mikołaj Steike, Michał Wichliński, Quirino Estrada, Krzysztof Przybył

**Affiliations:** 1 Faculty of Mechanical Engineering, Institute of Machine Design, Poznan University of Technology, Piotrowo 3, 60-965 Poznan, Poland; wiktor.lykowski@doctorate.put.poznan.pl (W.Ł.); mikolajsteike@gmail.com (M.S.); 2 Department of Renewable Energy Sources Engineering and Technical systems, Faculty of Mechanical Engineering, Bydgoszcz University of Science and Technology, Al. Prof. S. Kaliskiego 7, 85-796 Bydgoszcz, Poland; werkru001@pbs.edu.pl; 3 Faculty of Infrastructure and Environment, Częstochowa University of Technology, ul. J. H. Dąbrowskiego 73, 42-201 Częstochowa, Poland; michal.wichlinski@pcz.pl; 4 Instituto de Ingenieria y Tecnologia, Universidad Autonoma de Ciudad Juarez (UACJ), Ciudad Juarez 32310, Chihuahua, Mexico; quirino.estrada@uacj.mx; 5 Department of Dairy and Process Engineering, Food Sciences and Nutrition, Poznan University of Life Sciences, Wojska Polskiego 31, 60-624 Poznan, Poland

**Keywords:** DEM, comminution, angle of repose, restitution coefficient, breakage probability, biomaterials

## Abstract

The strength and energy of processed biological materials depend, among others, on their properties. Despite the numerous studies available, the relationship between the internal structure of corn grains and their mechanical properties has not yet been explained. Hence, the aim of the work is to explore the relationship between the internal composition of maize kernels and its mechanical properties by studying the impact of the maize seed coat thickness on its breakage susceptibility. To achieve the assumed goal, selected physical properties (length, width, and thickness) of corn grains were distinguished, and a static compression test was carried out on the Insight 50 kN testing machine (MTS Systems Corporation, Eden Prairie, MN, USA) with a test system for experimental verification of the compression behavior of biological materials. Furthermore, after the compression test, the thickness of the seed coat was measured using a laboratory microscope. It was found that there is a correlation between the thickness of the maize seed coat and force, deformation, and mass-specific energy at the bioyield point. The presented data constitute a foundation for the development of a mechanistic breakage model considering the variable strength properties of the seed coat and endosperm as the structural elements of kernels. Further research should be focused on the determination of the strength properties under dynamic conditions and revealing the relationship between the loading rate, strength properties, and internal structure for several maize varieties, which better reflect the ranges of variability in the real nature of mechanical processing.

## 1. Introduction

Corn occupies the largest area of cultivation in the world and is the most commonly processed grain material [1,2]. The United States, in 2021, produced about five times more corn kernels than the European Union [2]. Corn kernels are one of the most frequently processed materials requiring many preparatory operations, e.g., drying, cleaning, sorting, grinding, etc. The crushing and extraction of corn grain are used in many areas of the agro-processing industry [3,4], where the size reduction plays the most important role. To initiate the breakage of material structure, it is necessary to provide an adequate amount of energy [5]. The mechanical properties of corn cobs, stalks, and grains determine the design and operating parameters of devices intended for cutting, harvesting, and processing (e.g., grinding). They also affect the energy consumption of cutting and crushing machines and devices [6]. The determination of the critical values of forces, stresses, and energy for kernel breakage is of key importance in the design of processing machines. This helps to achieve the assumed design values of the energy and environmental efficiency indicators of the machines. Moreover it can be used in the modeling of machines and processes involving numerical methods, i.e., the discrete element method (DEM) [6]. Knowledge of the mechanisms of material behavior under the influence of applied loads is strategic when modeling and predicting the fracture phenomenon [7,8]. The strength and energy necessary to divide the structure of processed biological materials depend, among others, on their properties and primarily on their chemical composition and internal structure [6,9]. The analysis of previous studies indicates that the energy of grain breakage depends, among others, on its size [10], moisture, strength [11], and maturity conditioned by the harvest period. The studies carried out on the example of wheat also indicate that the energy necessary to shear this grain increases with increasing grain mass and vitreousness [12]. An important factor influencing the value of the breakage energy is the grain temperature, in particular the temperatures higher and lower than the room temperature [12]. The studies conducted by Romanski and Stopa [13] on shearing wheat grains have shown that the shear energy increases with the grain dimensions during vertical and horizontal loading. Furthermore, they found that the shear energy changes non-linearly with the increase in moisture content [13]. Initially, it was observed that the energy increases and then decreases with increasing water content in the grain structure [13]. The analyses presented in previous studies [14,15] have shown that moisture modifies not only the strength and energy of grain breakage, but also affects its shape, size, and bulk density. Therefore, the variability in material properties have an influence on the breakage probability and the distribution of grain sizes after crushing under specified loading conditions [16]. Several studies have described the strength and breakage pattern for brittle materials [17], including the characteristics of breakage probability [18]. For biomaterials, including corn, the visible biodiversity [19], even within one species [20], makes it difficult to model the breakage process with a high accuracy and consequently develop the design of processing machines and devices with a high size reduction efficiency and low energy consumption. Only in a few studies [21,22,23,24], has the breakage behavior been described in terms of breakage probability. Those include the influence of factors such as the internal structure, moisture, or grain size on the fracture pattern and mechanical properties [21,22,23,24].

Most of the available studies focus on determining the resistance to crushing and determining the hardness of kernels, which affects the mechanical properties and the material response on the applied load [25]. The most important properties that influence the grain hardness were indicated as the chemical composition and starch content, the share of soft endosperm to hard endosperm, and moisture content [25,26,27,28,29]. Both soft and hard endosperms in corn grains are characterized by different internal structures and the degree of starch cell packing, which result in their different mechanical properties [30,31].

The seed coat is an important part of the kernel, protecting the internal structure against the environmental factors and damage, and also mechanical damage that can appear during processing [32]. The mechanical properties of the seed coat and its role in the breakage behavior was studied mainly for legume seeds, wheat, and rye [32,33,34,35,36,37]. Dobrzański and Szot [37] investigated the effect of the moisture content on the deformability and tension resistance of three different pea cultivars. In another study, Dobrzański and Szot [35] found that the seed coat strength is dependent on the moisture content, and the increase in seed coat thickness causes the increase in the force at breakage of soy bean, pea, and lentil seeds. The effect of the seed coat thickness on the seed hardness was also studied for lupine, pea bean, vetch (Szelejewska), field pea, wheat, and rye by Frączek et al. [34]. They showed that the seed coat thickness could be the predictor of kernel hardness with the inclusion of other factors, such as the moisture content in the regression equation. Mabille et al. concluded that the increase in deformation to fracture of seed coats was correlated with the moisture content and bran size differences after milling [36]. The studies conducted by Hebda and Frączek showed that the seed coat is important in the course of the fracture of biomass grains (soybeans) [33]. They indicated that the outer layer of the grains is more flexible and is responsible for transferring loads and bonding the structure [33]. An important observation from the research of Hebda and Frączek [33] is that grains can be treated as thin-walled vessels filled with a material of a certain viscosity. The transfer of loads in the grain structure is conditioned by the relations of the aforementioned structural elements. The strength model of maize grains should therefore include the structural variability in the loaded cross-section.

The literature review showed that there is a lack of available knowledge on the effect of seed coat thickness on the mechanical properties of maize kernels. The aim of this study was to assess the effect of the seed coat thickness on the strength properties of maize kernels for the purpose of developing a strength model that takes into account the strength variability in the seed coat and endosperm. The static compression test was used to examine the mechanical properties of maize kernels such as hardness, toughness, force, deformation, and specific energy at the bioyield point and at fracture. Next, the seed coat thickness and hard, vitreous endosperm was measured for the compressed samples under a microscope. Moreover, the theoretical apparent surface area of the seed coat and vitreous endosperm, theoretical share of the surface area of the seed coat and vitreous endosperm in the compressed cross-section, theoretical volume of the seed coat and vitreous endosperm, and theoretical share of the volume of the seed coat and vitreous endosperm in the volume of grain were calculated on the basis of the measurement. The correlation and regression analyses were used to assess the effect of the parameters connected with the thickness of the seed coat and hard endosperm on the maize kernel mechanical properties.

## 2. Materials and Methods

### 2.1. Sample Preparation

**Figure 1:**
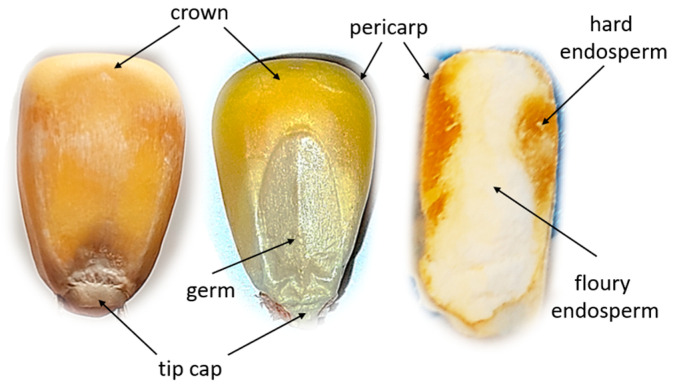
The internal structure of a corn kernel.

The research material consisted of the Celong variety of corn seeds. The selected maize is a medium-late variety (FAO 250, according to the COBORU Research Centre for Cultivar Testing classification [38]) grown for silage, characterized by a high grain yield, early flowering, and good resistance to water shortages [39]. To obtain the research material, first, Celong F1 corn seeds (seeds of hybrid varieties) were purchased from the main seed warehouse of TopFarms Seeds, Production Plant in Runowo, located in Wielkopolska Province. Then, corn was grown in experimental fields in the Szamotuły region, i.e., in the first corn cultivation region with the most favorable conditions for corn growth of corn for grain and silage, giving the greatest chance for full maturity of the plants in the growing season [38]. The sowing was carried out in the second half of April 2022, when the temperatures reached more than 10 °C. The corn seed density was 85,000 pieces per hectare, and fertilization with mineral fertilizers was applied in doses of 130 kg/ha of Urea and 200 kg/ha of Polifoska. A maize grain consists of 82% endosperm, approximately 12% is the embryo, and the remaining part is the root segment and the seed coat [40,41]. Figure 1 shows a cross-section of the grain illustrating its morphological structure.

The endosperm in maize occurs in two forms, namely floury endosperm and vitreous endosperm, which differ in structure [42]. Floury endosperm is composed of loosely packed starch grains, while vitreous endosperm is composed of densely packed starch chains with a crystalline structure. The starch chains comprise amylopectin and amylose and are embedded in a protein matrix. In addition, maize grains contain crude ash, oil, and fiber [43]. The seed coat constitutes a small percentage of the total grain structure and is composed mainly of cellulose and hemicellulose [44].

Based on the announcement of the Marshal of the Sejm of the Republic of Poland on the legal protection of plant varieties of 22 January 2021 (Journal of Laws of 2021, item 213) and the breeder’s declaration, the indicated Celong maize variety is legally protected by the breeder, but the authors received permission to use the grains for research. With the consent of the breeder, the authors could use the indicated plant material only for scientific research purposes, including testing, among others, the crushing force and seed coat thickness. The corn cobs for the research were handpicked in the 3rd week of October 2022 at a moisture content of 22%, pre-dried in the ambient temperature, and delivered in whole to the laboratory. The corn grains were then separated from their cobs and subjected to preliminary cleaning. A representative general sample for the research, weighing 1 kg, was taken in accordance with the PN-EN ISO 24333:2012P standard [45]. The grains were conditioned in a KBK-65W climatic chamber (Wamed, Warsaw, Poland) with forced air circulation for 48 h at a temperature of 20 °C to stabilize the moisture content. After the conditioning process, the dimensions and shape were measured as described in detail in Section 2.2, and the moisture content was measured according to the procedure described in Section 2.3. To prevent moisture loss, the samples were kept in sealed bags in the refrigerator before the compression test and between the compression test and measurement of the seed coat and endosperm thickness. The samples were taken out at least 16 h before the experiments in order for them to reach the ambient temperature.

### 2.2. Particle Size and Shape Characterization

To determine the size and shape, particle size analysis was performed using the Camsizer (Retsch GMBH, Haan, Germany) in accordance with ISO 13322-2:2006 [46]. As a result, a cumulative particle size distribution was obtained.

Next, 50 corn grains were selected, and their dimensions (length (*L*), width (*W*), height (*T*)) were measured using an electronic caliper with an accuracy of 0.01 mm, and their mass was measured using the AS 60/220.R2 PLUS laboratory scale (Radwag, Radom, Poland) with an accuracy of 0.1 mg. The selected grains were then subjected to compression tests.

Based on the measured values, three basic shape characteristics were calculated: sphericity index *f* (Equation (1)), flatness ratio *FR* (Equation (2)), and aspect ratio *AR* (Equation (3)) [6,15,29].

(1) $$f=\frac{{\left(L\cdot W\cdot T\right)}^{\frac{1}{3}}}{L}$$

(2) $$FR=\frac{T}{W}$$

(3) $$AR=\frac{W}{L}$$

### 2.3. Corn Seed Moisture Measurement

Corn stored in brick warehouses or silos typically has a moisture content ranging from 7% to 15% [1,37]. To characterize the selected research material, the moisture content was measured. Moisture testing was performed using a HR83 Halogen meter (Mettler-Toledo, Warsaw, Poland) with an accuracy of 0.001%. The moisture content of the tested samples was 11.57%.

### 2.4. Strength Testing Procedure

**Figure 2:**
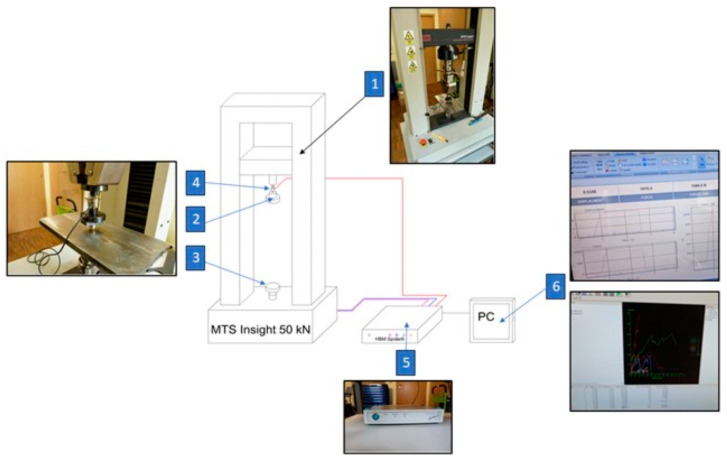
Schematic diagram of the test stand for measuring displacement and crushing force: 1—MTS universal tester, 2—upper compression plate, 3—lower compression plate, 4—U9B 1 kN sensor, 5—Spider 8 measuring amplifier (Hottinger Baldwin Messtechnik GmbH, Darmstadt, Germany), 6—laptop with Catman Easy data acquisition software, Version 5.0.2 (Hottinger Brüel & Kjær, Naerum, Denmark).

The tests were carried out using the MTS Insight Universal Testing Machines (MTS Systems Corporation, Eden Prairie, MN, USA) with a force capacity of 50 kN, equipped with a strain gauge sensor for force measurement and a displacement transducer, both having 0.5 accuracy classes (Figure 2). A testing sample consisted of 50 corn grains. The prepared grains, one by one, were held between compression plates (marked as 2 and 3 in Figure 1). The stand was connected to the PC (as shown in Figure 2) using Catman Easy data acquisition software, version 5.0.2 (Hottinger Brüel & Kjær, Naerum, Denmark), which enabled the recording and archiving of the force value applied to the sample as a function of its displacement. The compressive force was measured in parallel by using the testing machine and an additional U9B tensile sensor (Hottinger Baldwin Messtechnik GmbH, Darmstadt, Germany) registering the force values to 1 kN. Simultaneously, the signals from the displacement and force sensors were converted to voltage signals and then transmitted to the Spider 8 measuring amplifier (Hottinger Baldwin Messtechnik GmbH, Darmstadt, Germany), from which the data were read by using the Catman Easy, Version 5.0.2 software (Hottinger Brüel & Kjær, Naerum, Denmark), which was installed on the portable computer. The output signal values were acquired and processed using a frequency of 100 Hz.

### 2.5. Measurement of the Seed Coat Thickness and Endosperm Thickness

**Figure 3:**
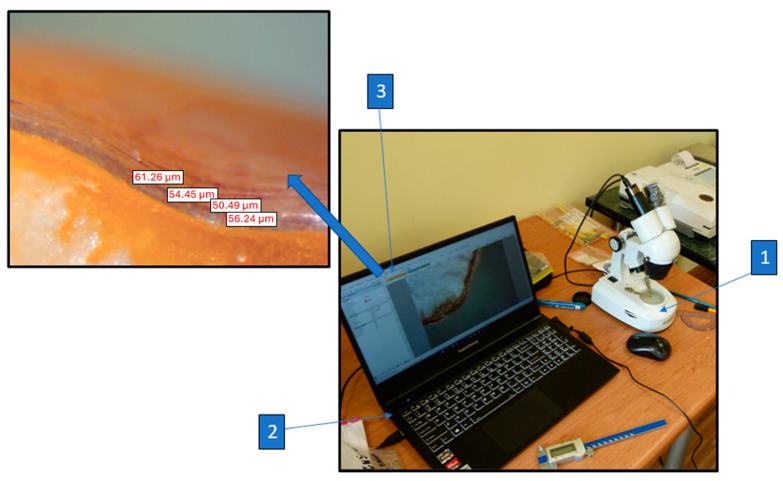
Schematic diagram of the research stand for measuring the seed coat thickness: 1—universal laboratory microscope (Bresser GmbH, Rhede Germany), 2—portable computer, 3—ToupView software, Version 3.7 (Photonics, Taipei City, Taiwan).

The thickness of the seed coat was measured after the compression tests for each kernel. The measurements were taken using a laboratory microscope(Bresser GmbH, Rhede Germany) with ×2 magnification and a SCMOS02000KPA 2.0MP 1/3.2 digital lens (Bresser GmbH, Rhede Germany). A diagram of the seed coat thickness measurement station is shown in Figure 3. Partially cracked and crushed samples after the crushing force test were cut open each time to create a plane necessary to measure the thickness of the seed coat. The use of a 2.0 megapixel digital lens (Bresser GmbH, Rhede, Germany) allowed for taking photographs of the seed cross-section and measurements of the seed coat thickness and vitreous endosperm thickness. The photographs obtained via the digital lens were imported into the ToupView software, version 3.7 (Photonics, Taipei City, Taiwan), where the seed coat $\bar{g}$ and vitreous endosperm $\bar{b}$ thickness were measured in the grain cross-section. Thickness measurements were taken at five measurement points at equal distances from each other. Then, the average thickness of the seed coat $\bar{g}$ and the thickness of the vitreous endosperm $\bar{b}$ in the grain cross-section were determined, as well as their apparent shares in the compressed cross-sectional area and grain volume.

For simplicity, it was assumed, taking into account the methodology for determining stresses in accordance with the ASAE S368.4 standard (American Society of Agricultural and Biological Engineers 2008) [47], that the area of the compressed cross-section *S* had the shape of an ellipse:

(4) $$S=\frac{\pi LW}{4}$$

where *L* and *W* represent the length and width of the kernel, respectively.

The apparent area of the seed coat *S_p_* in the compressed cross-section was calculated based on the average thickness of the seed coat $\bar{g}$ as the difference between the size of the compressed cross-section *S* and the cross-section of the grain interior without the average thickness of the coat:

(5) $$S_{p}=\frac{\pi LW}{4}-\frac{\pi \left(L-\bar{g}\right)\left(W-\bar{g}\right)}{4}$$

where *S_p_* is the apparent area and $\bar{g}$ is the average thickness of the coat.

The apparent area of the vitreous endosperm *S_b_* was determined similarly, but in addition to the average thickness of the coat $\bar{g}$, the average thickness of the endosperm $\bar{b}$ was included in Equation (5). The share of the vitreous endosperm or seed coat in the compressed cross-section was determined as the quotient of the apparent area of the vitreous endosperm *S_b_* or the apparent area of the seed coat *S_p_* to the total area of the compressed cross-section *S*, and this value was expressed as a percentage. Similarly, the share of the seed coat and vitreous endosperm in the volume of grain *V* was determined. The volume of the grain was calculated on the basis of its dimensions, assuming that the shape of the grain is similar to an ellipsoid.

(6) $$V=\frac{4\pi \frac{L}{2}\cdot \frac{T}{2}\cdot \frac{W}{2}}{3}$$

where *V* is the corn kernel volume and *L*, *T*, *W* represent the kernel dimensions, i.e., length, thickness, and width, respectively.

Then, the apparent volume of the seed coat *Vp* is equal to:

(7) $$V_{p}=V-\frac{4\pi \left(\left(\frac{L}{2}-\bar{g}\right)\cdot \left(\frac{T}{2}-\bar{g}\right)\cdot \left(\frac{W}{2}-\bar{g}\right)\right)}{3}$$

and the apparent volume of vitreous endosperm *Vb* is equal to:

(8) $$V_{b}=V-V_{p}\frac{4\pi \left(\left(\frac{L}{2}-\bar{b}\right)\cdot \left(\frac{T}{2}-\bar{b}\right)\cdot \left(\frac{W}{2}-\bar{b}\right)\right)}{3}$$

where *V* is the corn kernel volume, *V_p_* is the apparent volume of the seed coat, *V_b_* represents the apparent volume of the hard endosperm, $\bar{g}$ is the average thickness of the coat, $\bar{b}$ is the average thickness of the hard endosperm, *L* is the kernel length, *T* represents the kernel thickness, and *W* represents the kernel width.

### 2.6. Determination of the Mechanical Properties of Corn Kernels

**Figure 4:**
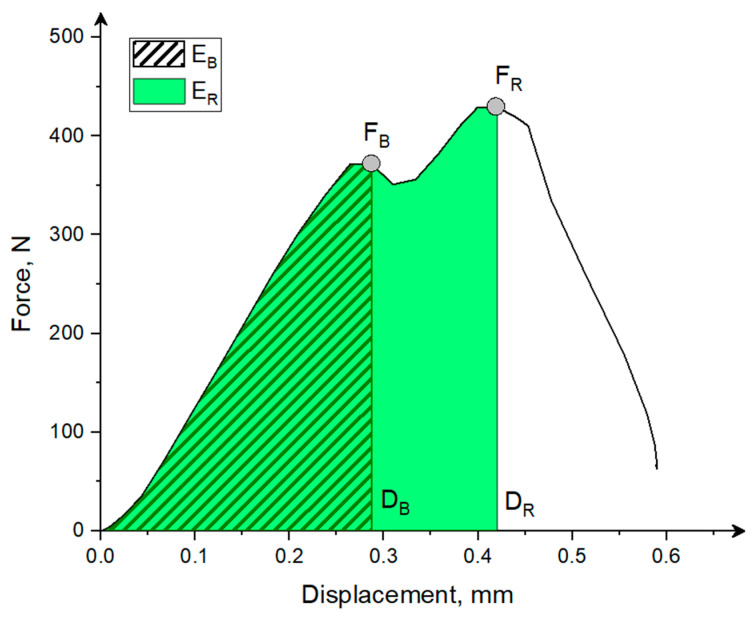
The typical force–displacement curve with marked bioyield force *F_B_*, rupture force *F_R_*, displacement to bioyield *D_B_* and rupture point *D_R_*, and energy to bioyield point *E_B_* and rupture energy *E_R_*.

Based on the results obtained from the compression test, the values of the force at the bioyield point, the breaking force, and the corresponding displacements, stresses, and energy values were determined in accordance with Figure 4.

The bioyield point in the literature is identified as a point on the force–displacement curve, which is the beginning, the first point of degradation of the grain structure. At this point, there is no increase in force or decrease in force with an increase in deformation [48]. The rupture force in this study was understood as the maximal force on the force deformation curve (Figure 4) that causes the external breakage and grain fragmentation [48,49]. The energy at the bioyield point *E_B_* and energy to induce breakage *E_R_*, equivalent in this case to the work done by the piston of the testing machine represented by the area under the force–displacement curve (as in Figure 4), was calculated as [6,50,51]:

(9) $$E_{\left(B,R\right)}=\int_{D_{0}}^{D_{\left(B,R\right)}}FdD$$

where *E* represents the energy needed, *F* is the force, and *D* is the displacement to reach the bioyield point and fracture point, marked in the subscript with the letters *B* and *R*, respectively.

The mass-specific energy, in turn, was expressed as the quotient of the energy to the bioyield point *E_B_*, the energy needed to induce fracture *E_R_*, and the mass of a single compressed grain [6,17]. In addition, the values of hardness and toughness of the kernels were determined, which are among the most important mechanical properties that influence the course of the fracture process. The hardness *H* was calculated as the ratio of the fracture force to the deformation caused by this force [52]:

(10) $$H=\frac{F_{R}}{D_{R}}\;\;\;\left[\frac{N}{mm}\right]$$

where *H* is the kernel hardness, and *F_R_* and *D_R_* represent the force and displacement at fracture, respectively.

The toughness was defined as the portion of energy absorbed until the fracture *E_R_* per unit volume *V* [52]:

(11) $$P=\frac{E_{R}}{V}\;\;\;\left[\frac{mJ}{mm^{3}}\right]$$

where *P* is the kernel toughness, *E_R_* is the energy needed to induce fracture, and *V* is the kernel volume.

### 2.7. Data Analysis

The basic descriptive statistic of data was performed at first, so the mean values, medians, standard deviations, standard error of means, variance, and upper and lower confidence intervals for the mean and range were calculated. Pearson correlation analysis was used to assess if there is a relationship between the thickness of the maize seed coat and share of hard endosperm in the kernel structure. The strength of the relationship between the variables was established based on the calculated values of the Pearson’s coefficients and the statistical significance assuming a 95% confidence level. In the interpretation of the correlation results, we used the Guilford scale (Table 1) [53]. According to the ASABE standard [47], for reliable mechanical properties estimation, the minimal sample size of 20 grains is recommended. In our study, we used 50 kernels to decrease the estimation error and sustain the experimental efficiency. As evidenced in different studies for plant materials, including maize, the sufficient sample size can vary between the hybrid, maturity, region, and the purpose of the analysis and should be established for the study context and goals [54]. Undoubtedly, increasing the sample size will increase the accuracy and precision of predictions, however simultaneously will decrease the experimental efficiency. According to the study of Toebe et al. [54], to detect weaker correlation, a bigger sample size is needed to detect strong correlations. Moreover, for a bigger sample size, the statistical significance is reached more often than in the smaller one [54]. Taking into account the high variability in the kernel properties and internal structure, detecting even small values of correlation coefficient will provide useful information about the significance of the variable in relation to the mechanical properties of the kernel. The relationships of practical importance were those with *r* ≥ 0.30 indicating the medium effect of the independent variable on the dependent variables [55]. The regression analysis was performed for the significant correlations between the variables to assess to what extent the thickness of the seed coat and endosperm (as well its estimated volume, area, and the share in the kernel structure) will explain the variability in the mechanical properties, such as hardness, toughness, force, deformation, and specific energy at the bioyield point and fracture point. The Levenberg–Marquardt algorithm was used to determine the mathematical expressions describing the relationship between the variables, assuming a 0.05 level of significance. The OriginPro software—version 2024 (OriginLab Corporation, Northampton, MA, USA) was used for the data analysis.

## 3. Results and Discussion

### 3.1. Particle Size and Shape

**Figure 5:**
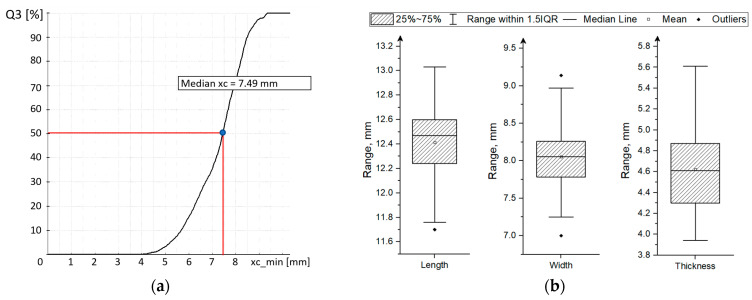
Results of particle size characterization (**a**) the cumulative size distribution (Q3) of maize kernels estimated based on the value of measured minimum chord diameter (xc_min); (**b**) the box plots of measured length, width, and thickness of 50 corn kernels selected for the compression test.

**Figure 6:**
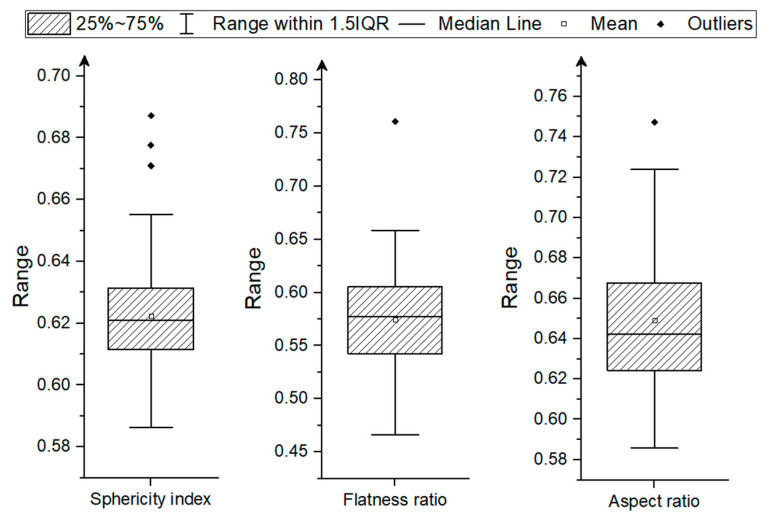
The shape characteristics of corn grains based on the single grain measurement.

Figure 5 and Figure 6 show the results of the size characteristics of Celong corn grains. The average width of the corn grains was 8.06 mm, the average length was 12.41 mm, and the average height was 4.62 mm. The average weight of a single grain was 0.32 ± 0.04 g, and the average grain volume calculated using Equation (6) was 242.96 ± 29.56 mm^3^. The average area of the theoretical compressed cross-section was 78.42 ± 5.31 mm^2^.

The corn grains used in the study were characterized by rounded edges and moderate sphericity according to the Krumbein scale [57]. The mean sphericity was 0.62 ± 0.02 and ranged from 0.586 to 0.687 (see Figure 6). The values of the flatness ratio (average 0.57 ± 0.05) and aspect ratio (average 0.65 ± 0.03) indicate irregularity and flattening of the grain in the plane determined by the thickness and width, and elongation in the plane determined by the width and length of the grain (see Figure 6). The lack of sphericity and the flattened shape of corn grains affect the behavior of grains under the influence of destructive loads, especially the stress distribution within the material and the direction of crack propagation. Usually, the grains with lower sphericity are more durable and more resistant to cracks than round, more spherical grains [25,58,59]. Furthermore, for particles of irregular shape, radial cracks propagating from the contact area were found to be caused by tensile stresses due to the action of components of the vertical and horizontal loading force [60].

### 3.2. The Share of the Pericarp and Hard Endosperm in the Corn Kernels

Table 2 presents the results of the measurements of the thickness of the seed coat and vitreous endosperm for the tested maize grains. The measurements carried out showed that the average thickness of the seed coat in the grain was 44.86 ± 3.71 μm, and its theoretical surface area was (1.83 ± 0.15)% of the total cross-sectional area of the grain. In turn, the theoretically determined volume of the seed coat was (3.74 ± 0.35)% of the total volume of the grain. The average thickness of the vitreous endosperm for the grains of the tested sample was 250.71 ± 103.59 μm, and its theoretical surface area was 9.18 ± 3.16 mm^2^, which is less than 12% of the total compressed cross-sectional area. Theoretically, the vitreous endosperm was on average (15.78 ± 7.26)% of the total volume of the maize grain. It should be emphasized that these are theoretical approximate values. In order to precisely determine the share of vitreous endosperm and the thickness of the seed coat, it would be necessary to use much more accurate and precise, but also much more expensive and time-consuming) research methods that allow for the differentiation and determination of the volume of layers of different density, e.g., microcomputed tomography.

Important from the point of view of further food, feed, or energy processing is the ratio of hard to soft (floury) endosperm, which defines the vitreousness of the kernel [61]. The vitreousness is correlated with the kernel hardness, and usually the more vitreous, the higher the hardness of the kernel [61]. Based on the vitreousness values, corn hybrids are classified as the floury (composed almost only of soft endosperm) dent (with soft endosperm closed from both sides by hard endosperm) and flint types (with a small proportion of soft endosperm), which differ in the content of the hard and soft endosperm [62]. For the corn grains tested, the theoretically determined share of vitreous endosperm to floury endosperm was 0.21 ± 0.12. The ratio of vitreous endosperm suggests that these are dent grains, with a share of vitreous and floury endosperm with a predominance of the floury part, which will determine, to some extent, the mechanical properties of the grains.

### 3.3. Mechanical Properties of Corn Kernels

The strength properties of grains are one of the basic parameters that are important for estimating the loads in processing machines, e.g., maximum loads to which grains can be subjected without causing damage to their structure that deteriorates their quality or estimating the energy demand of crushing machines. Table 3 presents the results of the tests of the selected mechanical properties for maize grains. The average value of the force at the bioyield point was 272.8 ± 116.63 N with an average displacement of the piston of the testing machine equal to 0.14 ± 0.09 mm at this point. The occurrence of the bioyield point was observed only for 22 kernels. In the case of the remaining grains, only a fracture point was detected, characterized by a rapid drop in force. The average value of the force that caused the grain fracture was 410.4 ± 222.12 N, and the average critical displacement for which the crack was observed was 0.28 ± 0.14 mm. The average energy input to reach the biological yield point was 89.89 ± 66.04 J/kg, and the average energy required for kernel breakage was almost three times higher (259.26 ± 201.39 J/kg). The corn grains were characterized by a hardness of 1722.83 ± 822.93 N/mm and a toughness of 0.34 ± 0.27 mJ/mm^3^.

### 3.4. The Influence of the Seed Coat Thickness and Vitreous Endosperm on the Strength Properties of Maize Grains

Corn grains are characterized by high structural variability in relation to a single corn variety, as indicated by the results presented in Table 4. The structural composition and in consequence the mechanical properties will vary also between varieties. The studies conducted on many different cereal grains have shown that structural variability is a significant factor influencing the strength properties [21,28,31]. The results obtained in our study are consistent with the observations made earlier by other researchers.

**Figure 7:**
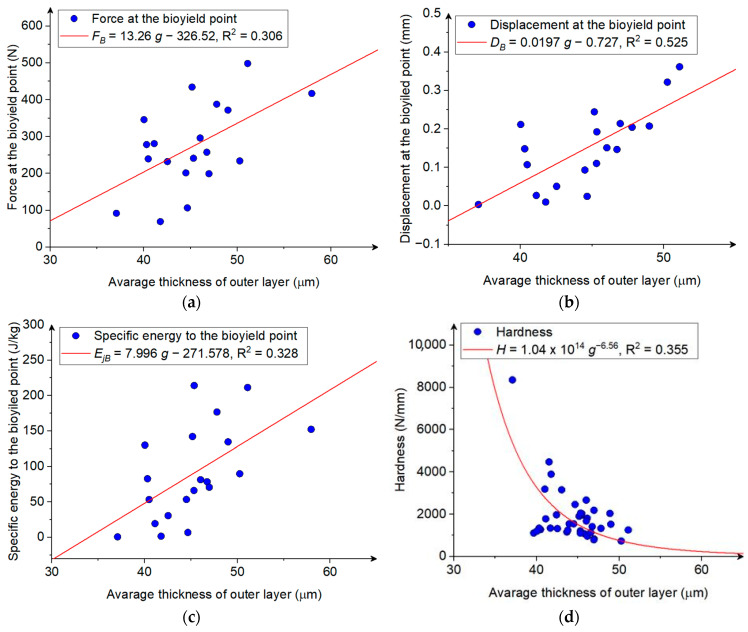
(**a**) The force at the bioyield point, (**b**) the displacement at the bioyield point, (**c**) specific energy at the bioyield point (**d**) hardness as a function of the average thickness of the seed coat in the maize kernel.

With the increase in the thickness (area and share in the surface and volume of the grain) of the seed coat, an increase in force was observed at the point of biological flow, which is confirmed by the statistically significant, moderate (0.40 ≤ *r* > 0.70), and high (0.70 ≤ *r* > 0.90) positive values of the correlation coefficients between the variables studied (Table 4). The variability in the force at the bioyield point was approximately 30% explained by the linear relationship of the seed coat thickness as presented in Figure 7a.

The increase in the thickness of the seed coat and the resulting derived thickness indices also caused an increase in the displacement at the bioyield point, as indicated by the moderate (0.40 ≤ *r* > 0.70) and high (0.70 ≤ *r* > 0.90) positive values of the correlation coefficients (Table 4). The variability in the kernel deformation at the bioyield point was 52.5% explained by the linear relationship of the seed thickness coat as presented in Figure 7b. The above relationships result from the strength properties of the seed coat and its internal composition. The seed coat of the maize grain is mainly composed of hemicellulose (∼67%), cellulose (∼23%), and lignin (∼0.1%) [44]. Both hemicellulose and cellulose are natural biopolymers that are characterized by a high tensile strength and flexibility [63,64,65]. In a related work, Zhang et al. demonstrated that cellulose chains in plant tissues can strengthen the plant cells, but at the same time, these chains can slide relative to each other, making the tissue elastic—elastic below the yield point and plastic above the yield point [65]. Therefore, the presence of the seed coat acts as a kind of elastic outer shell that holds the interior of the material. Similar observations were made in the work published by Hebda and Frączek [33]. The increase in the thickness of the seed coat causes an increase in forces up to the biological yield point of the grain due to the increase in the strength of the seed coat, especially against the tensile stresses that occur in cross-sections perpendicular to the compressed cross-section [66]. At the same time, due to the high elasticity of hemicellulose and cellulose fibers that make up the seed coat [63,64], a greater deformation capacity is observed up to the bioyield point. However, only a slight influence of the surface and volume of the seed coat on the values of deformation at the fracture point is observed, as indicated by the low (0.20 ≤ *r* > 0.40) positive values of the correlation coefficient (Table 4). The increase in the thickness of the seed coat and the associated increase in forces and displacements at the bioyield point cause an increase in the mass-specific energy at this point, as indicated by positive statistically significant moderate (0.40 ≤ *r* > 0.70) correlation coefficients (Table 4). The variability in the kernel deformation at the bioyield point was 32.8% explained by the linear relationship of the seed thickness as presented in Figure 7c. The increase in the thickness of the seed coat, and resulting increase in the share of the seed coat in the grain volume and in the compressed cross-section of the kernel results in the increase in flexibility and resistance to tensile stress, which leads to a decrease in hardness (moderate negative correlation (0.40 ≤ *r* > 0.70), Table 4). The relationship between the hardness and thickness of the seed coat can be represented by a power function (Figure 7d), which explains 35.5% of the variability in the dependent variable. This is consistent with the results obtained by Frączek et al. [34]. They found that the hardness of lupine, pea, bean, vetch, field pea, wheat, and rye changes exponentially with the seed coat thickness; however, the seed coat cannot be the only factor determining the seed hardness [34].

**Figure 8:**
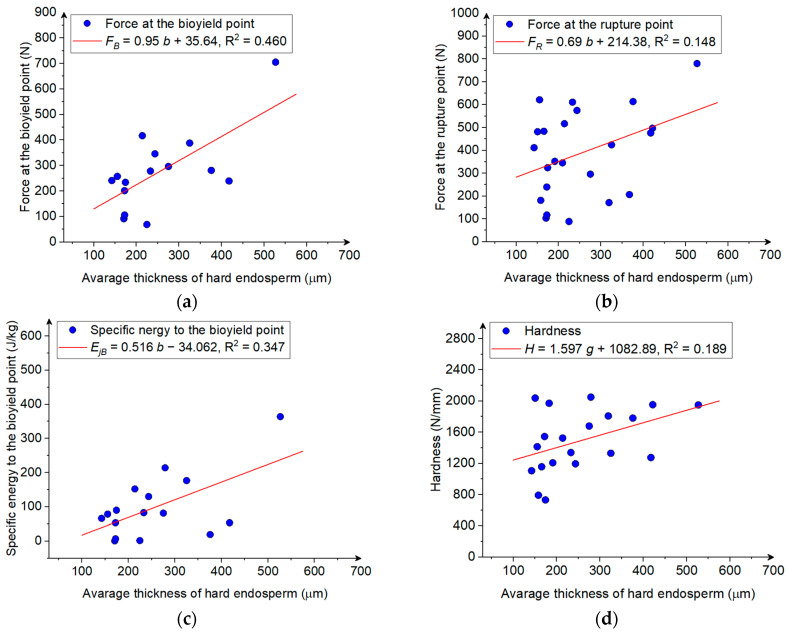
(**a**) The force at the bioyield point, (**b**) the force at the rupture point, (**c**) the mass-specific energy at the bioyield point, (**d**) the hardness as a function of the average thickness of the hard endosperm in the corn kernel.

The thickness, apparent area, and apparent volume of the vitreous endosperm in the maize grain had the greatest effect on the force and mass-specific energy at the bioyield point, as indicated by a statistically significant positive correlation (*r* > 0.56, Table 4). Weak positive correlations (0.20 ≤ *r* > 0.40) were also observed between the area of the vitreous endosperm and its share in the compressed cross-section and the force causing kernel fracture and displacement at the bioyield point (Table 4). The vitreous endosperm is one of the kernel’s structural components, along with the floury endosperm and the seed coat. In contrast to the floury endosperm, the vitreous endosperm is characterized by a greater strength and a more compact internal structure: the starch particles are more tightly packed [28,31,62]. Studies have shown that fracture begins in the floury endosperm with the lowest strength and spreads further to the outer layers [31]. The increase in the thickness of the vitreous endosperm naturally increases the fracture propagation path and causes greater forces to be required to break the internal bonds. This leads to an increase in the force to the bioyield point (Figure 8a), and consequently to an increase in the rupture force (Figure 8b). The variability in the force at the bioyield point was 46% explained by the linear dependence on the hard endosperm thickness (Figure 8a), while the variability in the rupture force was only 14.8% explained by the linear changes in the hard endosperm thickness (Figure 8b).

The Increase In the thickness of the vitreous endosperm causes an Increase In the mass-specific energy to the bioyield point. This is connected with the increase in the force at the bioyield point, when the share of the hard endosperm increases. The increase in the hard endosperm explained about 35% of the variability in the mass specific energy (Figure 8c). The increase in the thickness and share of the hard endosperm in the corn kernel structure also caused an increase in the kernel hardness (Table 4, Figure 8d). Similar findings can be found in the studies [25,27,28], which report that the share of the vitreous endosperm and its proportion with respect to the soft endosperm highly influence the grain hardness. The vitreous endosperm is characterized by densely packed starch cells embedded in the protein matrix. The starch structure in the endosperm is crystalline, which makes the kernel more resistant to damage and less deformable, as the starch chains cannot freely slide against each other [25]. According to the results presented by Gao et al. [28], the increased content of the protein in the endosperm will increase the kernel hardness.

The obtained values of correlation coefficients indicate the occurrence of mainly moderate and high correlations according to Gilford’s scale (Table 1) between the independent and dependent variables, which in most cases have practical significance—the value of correlation coefficients *r* was greater than 0.3. It should be stated that these are values confirming the existence of a relationship between the thickness of the seed coat, thickness of the hard endosperm, and the considered mechanical properties taking into account the large dispersions of values of the mechanical properties obtained for maize grains and the sample size tested. The regression models explained the variability in the mechanical properties of maize grains to a moderate degree (R^2^ below 0.55, mostly in the range 0.15–0.35) depending on the thickness of the seed coat and hard endosperm. However, taking into account the share of the seed coat (3.74%) and hard endosperm (15.78%) in the entire grain volume, it can be said that these are quite high values. Individual variables are therefore not sufficient, but important predictors of changes in the mechanical properties of maize. This is influenced by the dependence of the mechanical properties of maize also on factors other than the thickness of the seed coat or endosperm, mutually dependent factors, which include, among others: the internal structure, the chemical composition (mainly shares and structure of starch and protein), grain maturity, the distribution of individual structural elements inside the grain, or moisture content. Therefore, taking into account the thickness of the seed coat, the thickness of the endosperm, and additionally other variable factors influencing the mechanical properties, it is possible to improve the prediction power of regression models. The above statements are consistent with the results obtained by Frączek et al. [34] for grains other than maize, where it was found that in addition to the seed coat thickness, other variable factors, e.g., moisture, should be taken into account in the prediction models.

The research results indicate a varied influence of the structural components on the maize kernel mechanical properties and damage behavior. Considering a kernel as a whole, the floury endosperm with the lowest strength will be damaged first under compressive loadings, and then the fracture will propagate to the outer layers [23,66]. The appearance of the visible damage that affects the kernel quality will be then dependent on the mechanical properties of the vitreous endosperm and seed coat. Each of the grain components is responsible for different mechanical properties; therefore, it can be said that the grain can be treated as a composite material composed of layers with different properties. This concept was proposed by, among others, Singh et al. [67] and Hebda and Frączek [33], who claimed that grains can be treated as a material with a certain viscosity enclosed within a thin-walled shell, which in the case of maize is the seed coat. Therefore, grain strength models should take into account the variable strength properties of the individual structural elements.

It should be emphasized that the share of structural elements and the chemical composition of the grain depend on the maize variety. The presented results will therefore reflect the influence of the thickness of the seed coat and hard endosperm for grains from the tested variety. For floury-type kernels, the share of the hard endosperm in the structure is smaller [62] which could affect both the values of the correlation coefficients and the values of the determination coefficients for the obtained regression models. Importantly, both the thickness of the seed coat and the thickness of the endosperm depend on the cultivation conditions and the maturity of the grains in the growing season [68]. Under European conditions, early and medium-early maize crops dominate, although the emerging medium-late varieties are adapted to the specific climatic conditions and also reach maturity under European conditions [68]. Grains of incomplete maturity may be characterized by smaller dimensions and have an incompletely developed endosperm, in which the starch cells and the protein matrix have not been fully formed and filled, which is why they are characterized by the smaller sizes of the cells that build them [69]. The thickness of the seed coat and the developed endosperm, and consequently their effect on the compression characteristics of maize grains, also depends on the fertilization and cultivation conditions, which will affect the growth rate and maturity of the grains [69]. From the point of view of the currently occurring water shortages during the growth period, the hydrological conditions can significantly affect the content of the seed coat and the vitreous and floury endosperm [70]. The studies conducted by Yu et al. [70], using the example of wheat grains, indicated that periods of drought affect the size of starch cells in the grain structure and cause a decrease in the grain weight, the share of starch in the grain, and a decrease in the amylose-to-amylopectin ratio.

The seed coat is responsible for ensuring the integrity of the grain structure, but also for protecting it from pests, diseases, and external factors, including mechanical damage [32]. Mechanical damage dominates during operations such as harvesting, transport, and shelling as a result of multiple collisions with the structural elements of the processing machines [71]. The results from our study indicate that the seed coat and the thickness of the hard endosperm have a significant effect on the mechanical properties of maize grains and should be taken into account in the modeling of processing machines together with the other material properties of this kernel. The results constitute a valuable knowledge base for designing the loads exerted on grains, e.g., in combines, sorters, shellers, and screw feeders. Based on the knowledge of grain strength, it is possible to select the speed and mass of the elements of machines that are in contact with grains, which helps to minimize grain damage resulting from excessive loads. Moreover, the results can be used for the improvement of the wet and dry milling processes in terms of both the operational parameters and design features of mills [72]. The research conducted by Mabille et al. [36] for wheat grains showed that the thickness of the seed coat is correlated with the energy demand in the milling process, but also with the size of the bran fraction. In this context, the achievements presented in our study can be used to properly select the geometric features of the milling assemblies of the mills, but also to calculate their energy demand.

The results contained within our study can also support and enhance the separation processes of the various nutritional components of corn kernels. As concluded by Garcia-Lara [42], the understanding of macro- and microstructural properties can improve the ability to use the corn in different industry sectors. The knowledge about the inter-relations between the structure and the shares of the different structural components of corn provides a better insight into the mechanical behavior during processing and constitutes a guide to properly understand the breakage, also in terms of seed layer separation with minimal energy expenditure.

## 4. Conclusions

The strength properties of maize grains are the basic input data in the design of processing machines. The structural variability in grains within a single variety is significant and affects the strength properties of grains. Studies have shown that both the thickness of the seed coat and the thickness of the vitreous endosperm affect the strength properties of maize. An increase in the thickness of the seed coat causes an increase in the force, grain deformation, and energy to the point of biological flow identified by the beginning of crack development, which is associated with the high tensile strength of the cellulose fibers that make up the seed coat. At the same time, an increase in the thickness and share of the seed coat in the grain causes a decrease in the hardness of the grain, due to the greater ability to deform (flexibility) provided by the cellulose fibers. An increasing share of the vitreous endosperm causes an increase in the value of the force and energy to the point of flow, but also in the strength of the grain determined by the force causing the crack. In contrast to the seed coat, the increase in the thickness and content of the vitreous endosperm causes an increase in hardness, which is influenced by the compact and packed structure of the starch particles that form the vitreous endosperm. The results obtained indicate that grains can be considered as multimaterial composite elements with diversified strength characteristics. The results presented will be the basis for the development of a grain strength model (mechanical fracture model) that takes into account the variable strength of the seed coat and endosperm as structural elements of the grain. These data constitute a new contribution, as they are missing from the available literature. Further studies should focus on determining the strength properties under dynamic conditions and revealing the relationships between the loading rate, strength properties, and internal structure for several maize varieties, which will more fully reflect the ranges of variability in the real nature of the grinding process.

## Figures and Tables

**Table 1:** Guilford’s scale of significant correlations [56].

Value of r-Pearson’s	Magnitude of Correlation
<0.19	There is almost no relationship between the variables
0.20–0.39	Low correlation between the variables
0.40–0.69	Moderate correlation and substantial relationship
0.70–0.89	High correlation and strong relationship
0.90–1.00	Very high correlation

**Table 2:** The share of the outer layer and the hard endosperm in the corn kernel.

Data	Mean	Standard Deviation	SE of Mean	Lower 95% CI of Mean	Upper 95% CI of Mean	Variance	Median	Range (Maximum—Minimum)
$\bar{g}$, μm	44.86	3.71	0.59	43.68	46.05	13.8	45.31	20.87
*S_p_*, mm^2^	1.43	0.13	0.02	1.39	1.48	0.02	1.43	0.75
(*S_p_*/*S*)∙100, %	1.83	0.15	0.02	1.78	1.88	0.02	1.83	0.69
*V_p_*, mm^3^	9.05	1.06	0.17	8.71	9.39	1.12	8.79	4.95
(*V_p_*/*V*) 100, %	3.74	0.35	0.06	3.63	3.86	0.12	3.77	1.91
$\bar{b}$, μm	250.71	103.59	20.72	207.95	293.47	10,730.58	214	384.33
*S_b_*, mm^2^	9.18	3.16	0.63	7.88	10.49	10.01	8.26	12.07
(*S_b_*/*S*)∙100, %	11.7	3.9	0.78	10.09	13.31	15.24	10.37	13.83
*V_b_*, mm^3^	38.75	19.45	3.89	30.72	46.79	378.47	30.85	70.67
(*V_b_*/*V*) 100, %	15.78	7.26	1.45	12.79	18.78	52.73	14.12	26.25
Ratio of hard to soft endosperm	0.21	0.12	0.02	0.16	0.26	0.01	0.17	0.46

$\bar{g}$—average thickness of the seed coat, *S_p(b)_*—theoretical, apparent surface area of the seed coat (*p*) and vitreous endosperm (*b*), (*S_p(b)_*/*S*)∙100—theoretical share of the surface area of the seed coat (*p*) and vitreous endosperm (*b*) in the compressed cross-section, *V_p(b)_*—the apparent volume of the seed coat (*p*) and vitreous endosperm (*b*), (*V_p(b)_*/*V*) 100—theoretical share of the volume of the seed coat *(p)* and vitreous endosperm (*b*) in the kernel volume, $\bar{b}$—average thickness of the vitreous endosperm.

**Table 3:** The results of the mechanical properties of corn kernels.

Data	Mean	Standard Deviation	SE of Mian	Lower 95% CI of Mean	Upper 95% CI of Mean	Variance	Median	Range (Maximum—Minimum)
*F_B_*, *n*	272.8	116.63	26.76	216.59	329.02	13,601.64	257.52	429.36
*F_R_*, *n*	410.4	222.12	35.57	338.4	482.41	49,339.37	359.04	874.44
*D_B_*, mm	0.14	0.09	0.02	0.09	0.18	0.01	0.15	0.36
*D_R_*, mm	0.28	0.14	0.02	0.23	0.32	0.02	0.3	0.47
*E_jB_*, J/kg	89.89	66.04	14.77	58.98	120.8	4361.77	80.01	213.49
*E_jR_*, J/kg	259.26	201.39	31.84	194.86	323.67	40,556.3	211.15	740.48
*H*, *n*/mm	1722.83	822.93	131.77	1456.07	1989.6	677,221.15	1525.27	3745.21
*p*, mJ/mm^3^	0.34	0.27	0.04	0.26	0.43	0.07	0.26	0.97

*F_B_*—force at the bioyield point, *F_R_*—force at the rupture point, *D_B_*—displacement at the bioyield point, *D_R_*—displacement at the rupture point, *E_jB_*—mass-specific energy (work) to the bioyield point, *E_jR_*—mass-specific energy causing the kernel rupture, *H*—kernel hardness, *p*—kernel toughness.

**Table 4:** Results of Pearson’s correlation analysis between thickness, volume, and area of the seed coat and vitreous endosperm and maize kernel strength properties.

	*F_B_*, *n*	*F_R_*, *n*	*D_B_*, mm	*D_R_*, mm	*E_jB_*, J/kg	*E_jR_*, J/kg	*H*, *n*/mm	*p*, mJ/mm^3^
$\bar{g}$, μm	0.55 *	0.16	0.72 *	0.22	0.57 *	0.14	−0.48 *	0.12
*S_p_*, mm^2^	0.46 *	0.2	0.71 *	0.27 #	0.47 *	0.16	−0.47 *	0.16
(*S_p_*/*S*)∙100, %	0.63 *	0.06	0.65 *	0.13	0.67 *	0.08	−0.4 *	0.05
*V_p_*, mm^3^	0.38	0.16	0.63 *	0.29 #	0.37	0.09	−0.41 *	0.07
(*V_p_*/*V*) 100, %	0.53 *	0.13	0.5 *	0.09	0.56 *	0.16	−0.29 #	0.16
$\bar{b}$, μm	0.68 *	0.38 #	0.43	0.07	0.59 *	0.27	0.43 #	0.25
*S_b_*, mm^2^	0.71 *	0.42 *	0.45 #	0.11	0.61 *	0.3	0.45 *	0.27
(*S_b_*/*S*)∙100, %	0.68 *	0.35 #	0.45 #	0.04	0.6 *	0.26	0.42 #	0.22
*V_b_*, mm^3^	0.66 *	0.4 #	0.42	0.1	0.56 *	0.26	0.41 #	0.24
(*V_b_*/*V*) 100, %	0.63 *	0.35	0.4	0.03	0.56 *	0.26	0.46 *	0.24
Ratio of hard to soft endosperm	0.67 *	0.37 #	0.41	0.04	0.59 *	0.28	0.45 *	0.26

*—significance at the 0.05 probability level; #—significance at the 0.1 probability level. $\bar{g}$—average thickness of the seed coat, *S_p(b)_*—theoretical, apparent surface area of the seed coat (*p*) and vitreous endosperm (*b*), (*S_p(b)_*/*S*)∙100—theoretical share of the surface area of the seed coat (*p*) and vitreous endosperm (*b*) in the compressed cross-section, *V_p(b)_*—the apparent volume of the seed coat (*p*) and vitreous endosperm (*b*), (*V_p(b)_*/*V*) 100—theoretical share of the volume of the seed coat *(p)* and vitreous endosperm (*b*) in the kernel volume, $\bar{b}$—average thickness of the vitreous endosperm, *F_B_*—force at the bioyield point, *F_R_*—force at the rupture point, *D_B_*—displacement at the bioyield point, *D_R_*—displacement at the rupture point, *E_jB_*—mass-specific energy (work) to the bioyield point, *E_jR_*—mass-specific energy causing the kernel rupture, *H*—kernel hardness, *p*—kernel toughness.

## Data Availability

The original contributions presented in the study are included in the article, further inquiries can be directed to the corresponding authors.
